# Thirty-Eight-Year Follow-Up of Two Sibling Lipoid Congenital Adrenal Hyperplasia Patients Due to Homozygous Steroidogenic Acute Regulatory (STARD1) Protein Mutation. Molecular Structure and Modeling of the STARD1 L275P Mutation

**DOI:** 10.3389/fnins.2016.00527

**Published:** 2016-11-21

**Authors:** Khalil Khoury, Elie Barbar, Youssef Ainmelk, Annie Ouellet, Pierre Lavigne, Jean-Guy LeHoux

**Affiliations:** ^1^Department of Pediatrics, Faculty of Medicine, University of SherbrookeSherbrooke, QC, Canada; ^2^Department of Biochemistry, Faculty of Medicine, University of SherbrookeSherbrooke, QC, Canada; ^3^Department of Obstetrics and Gynecology, Faculty of Medicine, University of SherbrookeSherbrooke, QC, Canada

**Keywords:** LCAH, spontaneous puberty, fertility, pregnancy, steroidogenic acute regulatory protein (StAR, STARD1), cholesterol, molecular structure, therapy

## Abstract

**Objective:** Review the impact of StAR (STARD1) mutations on steroidogenesis and fertility in LCAH patients. Examine the endocrine mechanisms underlying the pathology of the disorder and the appropriate therapy for promoting fertility and pregnancies.

**Design:** Published data in the literature and a detailed 38-year follow-up of two sibling LCAH patients. Molecular structure and modeling of the STARD1 L275P mutation.

**Setting:** University hospital.

**Patients:** Patient A (46,XY female phenotype) and patient B (46,XX female) with LCAH bearing the L275P mutation in STARD1.

**Interventions:** Since early-age diagnosis, both patients underwent corticoid replacement therapy. Patient A received estrogen therapy at pubertal age. Clomiphene therapy was given to Patient B to induce ovulation. Pregnancies were protected with progesterone administration.

**Main Outcome Measures:** Clinical and molecular assessment of adrenal and gonadal functions.

**Results:** Both patients have classic manifestations of corticosteroid deficiency observed in LCAH. Time of onset and severity were different. Patient A developed into a female phenotype due to early and severe damage of Leydig cells. Patient B started a progressive pubertal development, menarche and regular non-ovulatory cycle. She was able to have successful pregnancies.

**Conclusions:** Understanding the molecular structure and function of STARD1 in all steroidogenic tissues is the key for comprehending the heterogeneous clinical manifestations of LCAH, and the development of an appropriate strategy for the induction of ovulation and protecting pregnancies in this disease.

## Introduction

Classical lipoid congenital adrenal hyperplasia (LCAH), which was originally described by Prader and Siebenmann (1957) is a severe form of congenital adrenal hyperplasia inherited as an autosomal recessive disease. Patients bearing this disorder show an impaired production of glucocorticoids, mineralocorticoids, and sex steroids. Their basal levels of adrenocorticotropin (ACTH) and plasma renin activity (PRA) are high with no steroidal response to ACTH, or human chorionic gonadotropin (hCG) treatment. Signs of adrenal insufficiency and hyperpigmentation occur in phenotypic female infants regardless of the karyotype (Bose et al., 1996).

In LCAH, the destruction in early fetal life of Leydig cells of 46,XY subjects, by the toxic effect of stored cholesterol and derivatives, eliminates testosterone biosynthesis and normal virilization. In such cases, external genitalia are female with blind vaginal pouch and absence of cervix, uterus, and fallopian tubes due to undamaged Sertoli cells producing the Müllerian inhibitory hormone. Occasionally reported development of Wolffian ducts testifies for the presence of testosterone synthesis early in fetal life (Ogata et al., 1989). When adequately treated, 46,XX subjects may undergo spontaneous puberty, feminization and even cyclic vaginal bleeding (Matsuo et al., 1994; Bose et al., 1997; Fujieda et al., 1997). Despite the presence of sufficient amounts of estrogens to produce secondary sexual development and endometrial growth, progesterone levels remain undetectable, indicating that the periodic vaginal bleeding is of a non-ovulatory nature (Fujieda et al., 1997). Thus, affected individuals are all phenotypically female with a severe salt-wasting crisis and hyperpigmentation and may die shortly after birth unless treated with steroid-replacement therapy (Hauffa et al., 1985). Non-classical LCAH has also been reported: Affected individuals present with a phenotype of late onset adrenal insufficiency with only mild or minimally disordered sexual development (Baker et al., 2006; Sahakitrungruang et al., 2010).

Because mitochondria from affected adrenal and gonads fail to transform cholesterol to pregnenolone, it has been postulated for many years that LCAH disease was due to a defect in the cholesterol side chain cleavage enzyme complex cytochrome P450 (P450scc) (Degenhart et al., 1972; Koizumi et al., 1977; Matteson et al., 1986) which is the first step in steroidogenesis: The conversion of cholesterol to pregnenolone. In searching for such defect, Lin et al. (1991), Sakai et al. (1994) and Fukami et al. (1995) found no mutations in the *CYP11A1* gene of affected individuals. However, mutations in CYP11A gene were subsequently reported resulting in indistinguishable clinical and hormonal phenotypes when compared to LCAH cases (Tajima et al., 2001; Katsumata et al., 2002; Hiort et al., 2005). In addition, these patients do not have the massive adrenal hyperplasia that characterizes LCAH (Miller, 2016).

With the discovery of STARD1 (STeroidogenic Acute Regulatory) protein (Clark et al., 1994), the leading cause of LCAH was then attributed to mutations in that protein (Lin et al., 1995; Bose et al., 1996). STARD1 is produced in the cytoplasm and mediates the biosynthesis of steroid hormones by controlling the transfer of cholesterol from the outer mitochondrial membrane (OMM) to the inner mitochondrial membrane (IMM) where P450scc is located (Farkash et al., 1986; Geuze et al., 1987). Moreover, numerous mutations disrupting STARD1 activity were found in the carboxyl-terminal of the protein (Lin et al., 1995; Bose et al., 1996; Bhangoo et al., 2005, 2006) suggesting that this is a biologically important domain. Another crucial domain for STARD1 is the cholesterol binding pocket which contains a putative salt bridge between Glu^169^ and Arg^188^ (Mathieu et al., 2002a). Other clinical STARD1 mutations are found at the binding site, in particular Glu^169^ and Met^225^, and they lead to LCAH (Miller, 1997).

In the absence of STARD1, steroidogenesis proceeds, for a temporary period, at about 14% of the STARD1 induced level (Lin et al., 1995; Tee et al., 1995; Bose et al., 1996; Miller, 1997; Khoury et al., 2006a). The pathogenesis of the different manifestations of LCAH involves STARD1-dependent and STARD1-independent steps of steroidogenesis (Bose et al., 1996; Miller, 1997). The STARD1-dependent phase would be the loss of the protein activity leading to a decrease of more than 80% of adrenal and gonad steroidogenesis. The STARD1-independent stage is characterized by a destruction of the steroidogenic capacity due to the accumulation of cholesterol, cholesterol esters and oxidation products. This engorges the cells and damages its cytoarchitecture through both physical displacement and biochemical actions.

We have previously communicated the cases of LCAH in two children (46,XX and 46,XY) of a French Canadian family due to a homozygous L275P STARD1 mutation (Khoury et al., 2004). Furthermore, ovulation and pregnancies in the 46,XX patient were possible with Clomiphene stimulation and progesterone administration during the first trimester (Khoury et al., 2006b, 2009). Here, we review data collected over a three-decade follow-up of these patients focusing on their clinical evolution, gonadal function, puberty and fertility. Finally, the STARD1 mechanism of action and the impact of the clinical mutants on the protein's structure/activity will be addressed. An informed consent form was signed by all participants for blood sampling and analysis, and studies were approved by the institutional “*Comité d'éthique de la recherche en santé chez l'humain du CHUS”*.

## Patient A (46,XY karyotype)

Patient A was born in summer 1977 after 41 weeks of normal gestation, weighing 3960 g and measuring 53 cm (Khoury et al., 2004). External genitalia were described as normal female phenotype with bilateral inguinal hernia. This was surgically repaired at the age of 5 weeks with no complications. Both gonads observed during surgery were pushed into the abdomen. At the age of 11 months, the patient suffered from gastroenteritis, hyperthermia, and dehydration. Blood pressure was 100/50, heart rate 120/min, and respiratory rate 24–28/min. Blood glucose was 3.8 mmol/L, sodium 121 mmol/L, chloride 93 mmol/L, potassium 5.4 mmol/L, and serum bicarbonate was 7 mmol/L. Intravenous fluid and glucose were administered to correct this situation. Four days after the cessation of the intravenous therapy, electrolytes disturbance and metabolic acidosis relapsed (sodium 126 mmol/L, chloride 103 mmol/L, potassium 6.3 mmol/L, sodium bicarbonate 14 mmol/L, and blood glucose 4.6 mmol/L). Intravenous rehydration was restarted. Physical examination was normal except for generalized and moderate hyperpigmentation, hypertrichosis on arms and on the lower part of her back. The external genitalia corresponded to those of a normal female with normal clitoris and vaginal orifice. There were no palpable gonads in the inguinal region and the scar of the previous surgical procedure appeared normal.

### Adrenal function

At the age of 11 months, serum cortisol was 640 nmol/L at 8 h 00 and 361 nmol/L at 16 h 00 (*N* = 165–635 nmol/L); serum aldosterone was respectively 641 and 319 pmol/L (*N* = 168–2570 pmol/L) and plasma renin activity was 11.9 ng/ml/s at 16 h (*N* < 4.17 ng/ml/s), and urine pregnanetriol 0.0 μmol/d (*N* = 0.06–0.6). Further investigations showed that serum cortisol, aldosterone as well as urinary pregnanetriol did not respond to intramuscular ACTH stimulation for 3 days (Table 1). Substitution therapy with mineralocorticoid (fludrocortisone acetate) and glucorticoid (cortisone acetate) was then started. Four months later, we decided to stop cortisone acetate to obtain more data on her glucocorticoid secretion.

**Table 1:** **Patient A**.

**Dates**	**Serum cortisol nmol/L (165–635)**	**Serum aldosterone pmol/L (168–2520)**	**Plasma renin ng/L/s (<4.17)**	**Urine pregnanetriol μmol/day (0.06–0.6)**
Day 2	966	670	19.46	0
Day 1	634	621	22.2	<0.03
Day 0	739	474	11.95	<0.03
Day 3	811	244	15.29	<0.03
Day 4	497	357	13.34	<0.09

*ACTH stimulation test: Injection of ACTH-Gel 25 IU/m^2^ /q 12 h/3 days During this test, the patient received NaCl 70 meq/d, the serum sodium was 120–131 mmol/L, potassium 6.0–6.9 mmol/L and urinary sodium 51–64 mmol/L, at 8 and 16 h respectively. Age of patient A, 11 months*.

For the following 20 months, the child was growing well (50 percentile for height and weight) and has no specific symptoms. From 35 to 41 months of age, the patient was admitted to the hospital for 3 episodes of hypoglycemia and acidosis during infectious illnesses. Blood glucose was 1.4–4.0 mmol/L, electrolytes were normal with high renin activity and elevated urinary sodium. Basal steroid hormones (while receiving fludrocortisone acetate 0.025 mg every 12 h) revealed low cortisolemia^*^ 69 and 8.28 nmol/L at 8 and 16 h respectively, normal aldosterone^**^ 737 and 208 pmol/L, 17 OH-progesterone 0.15 nmol/L (*N* = 0.1–2.7), DHEA 0.69 nmol/L (*N* = 0.38–2.52), DHEA-S 0.54 μmol/L (*N* = 0.13–0.54) testosterone 0.1 nmol/L (*N* = 0.07–0.9), and ACTH 33 pmol/L (*N* = 2–11). ACTH stimulation test was repeated at that time (intramuscular injections of ACTH-Gel 25 IU/m^2^ every 12 h × 5 days) under replacement therapy with fludrocortisone acetate 0.025 mg every 12 h and Dexamethasone 0.25 mg every 8 h. The higher values of blood cortisol and aldosterone obtained during this stimulation was 19.3 and 263 pmol/L respectively, urinary free cortisol^***^ was < 5.5 nmol/day and pregnanetriol^****^ 0.11 μmol/day at that moment. The result of previously ordered blood karyotype revealed the suspected 46,XY. Then clinical, biochemical and genetic studies confirmed the suspected diagnosis of what was believed at that time as 20,22 desmolase deficiency. Substitution therapy with fludrocortisone acetate and hydrocortisone continues, doses were regularly adjusted according to clinical symptoms, blood glucose and electrolytes as well as serum level of ACTH and PRA in addition to observations of growth and bone maturation.

Normal basal values from 2–5 years:

^*^ Cortisol 166–525 nmol/L (mean Δ after ACTH stimulation 400 nmol/L)
^**^ Aldosterone 83–971 pmol/L (mean Δ after ACTH stimulation 500 pmol/L)
^***^ Urinary free cortisol 8.28–24.8 nmol/d
^****^ Urinary pregnanetriol < 1.5 μmol/d

### Radiological investigations

At the moment of her first admission, at 11 months of age, the IV pyelography was normal. Several pelvic and abdominal echographies were performed from the age of 3 6/12 to 13 years. These showed no uterus, no visible gonads and no adrenal hypertrophy, the bone age was always significantly delayed (growth curve not shown).

### Gonadal function

FSH, LH and different androgens were measured at regular intervals from the age of ~3 years until the patient was 13 year old (Table 2). As can be seen, the levels of serum androstenedione, dehydroepiandrosterone, total and free testosterone were low to not detectable. hCG stimulation test (intramuscular injections 4000 IU/day for 5 days) was performed at the age of 13 years (while the patient was receiving fludrocortisone acetate and Dexamethasone). As shown in Table 2, hCG stimulation did not increase circulating levels of 17 OH-pregnenolone, 17 OH-progesterone, DHEA, androstenedione, and total and free testosterone.

**Table 2:** **Patient A**.

**Hormones age**		**FSH IU/L**	**LH IU/L**	**17 OH-Preg nmol/L**	**17 OH-Prog nmol/L**	**DHEA nmol/L**	**Andro nmol/L**	**Testo (total) nmol/L**	**Testo (free) nmol/L**
~3 years		–	–	−	0.15	0.69	−	0.1	–
~4 years		–	–	−	−	<0.35	<0.3	<0.35	–
5 10/12 years		3.9	<2.7	−	−	1.73	<0.3	<0.35	–
6 7/12 years		13.8	<5	−	−	2.08	<0.7	<0.35	–
7 6/12 years		8.6	<5	−	−	−	−	<0.35	–
8 years		23	<5	−	−	−	−	<0.35	–
8 9/12 years		11.8	5.1	−	−	−	−	<0.35	<0.6
10 4/12 years		11.6	<2	−	−	−	−	<0.35	<2
12 6/12 years		4.6	<0.5	−	−	<1.7	<0.3	<0.7	<3
hCG (IM) 4000 IU/dX5 days	D 0	33.2	1.8	<0.16	<0.3	<1.5	<0.3	<0.7	<1.0
	D 3	–	–	<0.16	<0.3	<1.5	<0.3	<0.7	–
	D 4	–	–	<0.16	<0.3	<1.5	<0.3	<0.7	–
	D 6	–	–	<0.16	<0.3	<1.5	<0.3	<0.7	<2.1

T, total; F, free; D, day; –, not available. Normal values are indicated for different ages:

*FSH: Prepubertal 0.26–3 IU/L, puberty Tanner II, III 0.6–10.9 IU/L*.

*LH: Prepubertal 0.02–0.3 IU/L, puberty Tanner II, III 0.2–5.0 IU/L*.

*17-OH pregnenolone: Puberty Tanner II, III 0.6–10.9 nmol/L*.

*17-OH progesterone: Prepubertal 0.09–2.7 nmol/L, puberty Tanner II, III 0.3–3.9 nmol/L*.

*DHEA: 2–5 years 0.38–2.52 nmol/L, 6–10 years 0.9–5.3 nmol/L, puberty Tanner II, III 0.87–10.5 nmol/L*.

*Androstenedione: 1–10 years 0.18–1.78 nmol/L, puberty Tanner II, III 0.6–2.87 nmol/L*.

*Testosterone (total): 3–10 years 0.07–0.66 nmol/L, puberty Tanner II, III 0.63–11.2 nmol/L*.

*Testosterone (free): Prepubertal 0.52–2.0 pmol/L, adult male 180–971 pmol/L*.

*Gonadal function of Patient A measured at different interval from age 3 to 13 years old*.

### Gonadal histology

**Figure 1:**
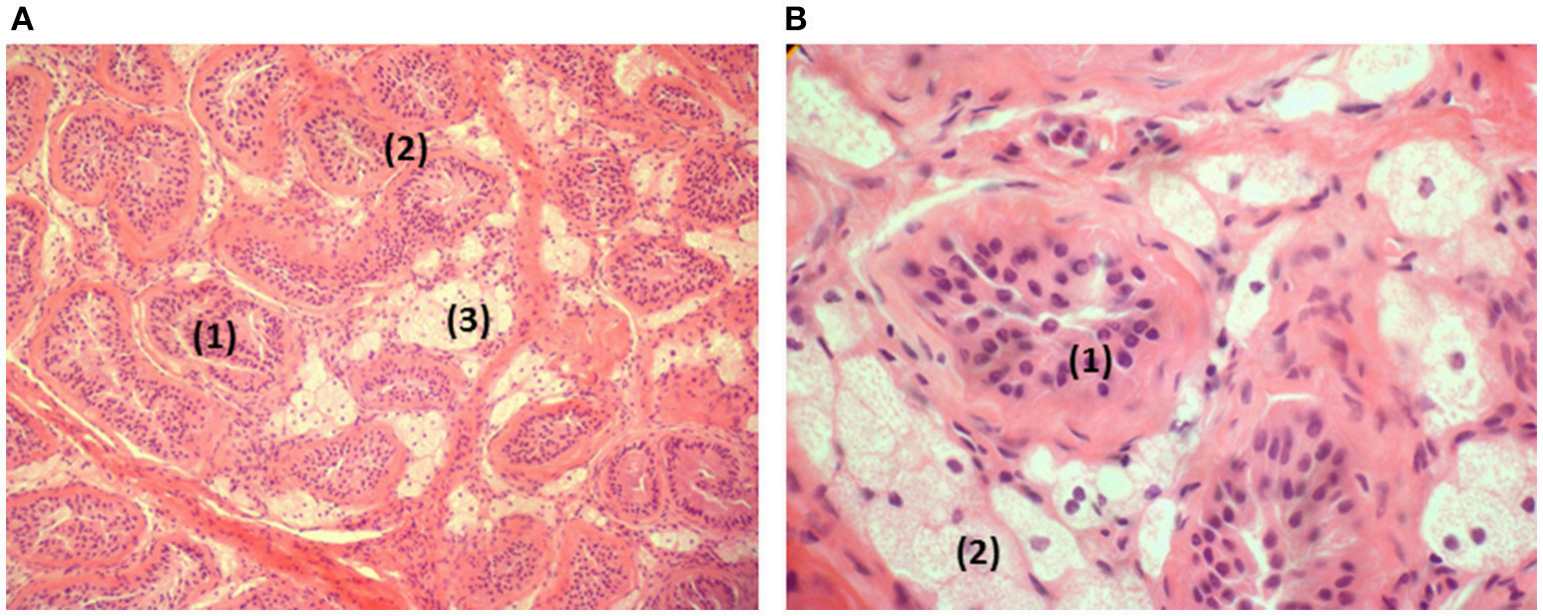
**Histology of the removed gonad. (A)** Testis in low magnification: atrophic seminiferous tubules (1). Hyaline thickening of the basal membranes (2). Hyperplasia and clarification of the Leydig cells (3). **(B)** Testis in higher magnification: atrophic seminiferous tubules (1) containing Sertoli cells in the absence of spermatogonia. Lipid overload of the Leydig cells (2).

A laparotomy was performed at the age of 13 5/12 years: The left gonad (3 × 1.5 × 1 cm) was removed. The exploration of the right side was negative and showed no Müllerian or Wolffian structures. The histology of the removed gonad is illustrated in Figure 1. Worth noticing is the atrophy and hyalinization of the seminiferous tubules, the presence of some germinal cells without spermatogonia, the presence of some Sertoli cells and the important engorgement of Leydig cells with fat.

### Growth

The patient's growth velocity decreased regularly from the age of 10 years until she was 14 year old. Bone age was always significantly delayed (curve not shown). The thyroid function and growth hormone secretions were normal. Progressive ethinyl estradiol therapy was started at the age of 14 leading to a gradual pubertal development (good breast development, moderate pubic hair) and an increase of the growth rate. Final adult height was in the normal mid-parental range.

## Patient B (46,XX karyotype)

Patient B was born in spring 1979 (Koizumi et al., 1977; Khoury et al., 2004). She had skin hyperpigmentation since the age of 1.5 months. At 4.5 months, she was hospitalized for fever, anorexia, fatigue and weight loss, and was treated with antibiotics (pharyngitis) for 10 days. Furthermore, she suffered from somnolence, vomiting and dehydration with hyponatremia and hyperkalemia (Khoury et al., 2009).

### Adrenal function

The endocrine investigations revealed a low/normal serum cortisol 174 nmol/L (*N* = 77–635), low/normal aldosterone 138.7 pmol/L (*N* = 166–1970) and renin activity 3.3 ng/L/s (*N* < 4.17). The urinary pregnanetriol was 0.59 μmol/day (*N* = 0.06–0.6). The ACTH stimulation (IM injections of ACTH-Gel 25 IU/m^2^ q 12 h. × 3 days) resulted of very small increases in cortisol (220 nmol/L) and aldosterone (277.4 pmol/L). She was growing well with no specific symptoms while treated only with fludrocortisone acetate 0.025 mg bid, up to the age of 15 months. From that age to 21 months she suffered 3 episodes of hypoglycemia during a varicella illness and otitis media. Her serum cortisol level was 8.28 nmol/L (*N* = 166–690), serum aldosterone and plasma renin activity were 141.3 pmol/L (*N* = 139–1498) and 0.67 ng/L/s (*N* < 2.8) respectively. Then Dexamethasone 0.25 mg p.o. every 8 h was added to fludrocortisone acetate before to undergo her second ACTH stimulation test. Results illustrated in Table 3 indicate a very low basal cortisol and incapacity of the zona fasciculata-reticularis to respond to ACTH stimulation; the same table shows low level of aldosterone with no response of the zona glomerulosa to ACTH stimulation. Consequently, the doses of glucocorticoids (Dexamethasone changed for hydrocortisone) and fludrocortisone acetate, were adjusted regularly during all the next years, according to her clinical evolution, blood glucose and electrolytes, as well as plasma ACTH and renin activity.

**Table 3:** **Patient B**.

**Time**	**Serum cortisol nmol/L (166–525)**	**Serum aldosterone pmol/L (139–1498)**	**Plasma renin ng/L/s (<2.8)**
Day 1	18.2	136	0.88
Day 0	5.52	161	6.47
Day 2	8.28	136	3.27
Day 4	13.8	180	5.36

*ACTH stimulation test: Injection of ACTH-Gel 25 IU/m^2^/12 h/3 days. Age of Patient B, 21 months. Normal values are in parentheses*.

### Gonadal function

The patient's serum levels^*^ of FSH and LH were normal up to puberty. Serum levels of 17 OH-Prog, DHEA, androstenedione, and testosterone were very low. Plasma ACTH varied from 43–4.6 pmol/L during that period. She started thelarche and pubarche at the age of 11 7/12 and 12 years, respectively. The hypophyso-gonadal function was previously summarized (Khoury et al., 2009). A spontaneous normal menarche started at the age of 14 2/12 years. The menstruations were regular with no dysmenorrhea. The basal temperature registered between 21 and 24 years indicated a monophasic non-ovulatory curve. Shortly after a Clomiphene stimulation, circulating 17β-estradiol level increased from 703 to 1428 pmol/L and progesterone level increased from 3.4 to 9.4 nmol/L (Khoury et al., 2004).

^*^Normal prepubertal values: FSH 1.0–4.2 IU/L, LH 0.02–0.18 IU/L, 17-OH progesterone 0.09–2.72 nmol/L, DHEA 0.4–6.6 nmol/L, androstenedione 0.28–1.75 nmol/L, testosterone 0.07–0.35 nmol/L.

### Radiological investigations

An abdominal and pelvic echography was performed at the age of 2 5/12 years. It revealed a normal uterus and ovaries. Adrenal glands have no particular aspect. This examination was repeated at 11 1/12 years and 12 3/12 years and showed a prepubertal uterus and normal ovaries with small follicles. A small delay of bone age was noted at different ages.

### Growth

Regular growth was observed. Peak height velocity began around the age of 13 years. Final adult height was in the normal mid-parental range.

### Fertility studies and childbirth

Clomiphene stimulation, to increase gonadal activities and induce ovulation, was started at the age of 25 years. First pregnancy occurred at the age of 25 4/12 years, unfortunately interrupted by spontaneous abortion 6 weeks later (Khoury et al., 2009). A second pregnancy (quadruple) was initiated later on with Clomiphene stimulation, and was protected with progesterone supplementation at the 17th day of the cycle. One fetus was naturally lost at 7 weeks of gestation and one feticide was done 1 ½ weeks later, so progesterone was continued up to the 25th week of gestation. In October 2005, two normal boys were born prematurely. This first child delivery was previously communicated in an international meeting in 2006 (Khoury et al., 2006b). At 28 years old, a third pregnancy initiated with Clomiphene stimulation, and progesterone was taken from the 17th day of the cycle up to the 17th week of gestation. After 36 weeks of normal gestation, a female child was born in October 2008 (Khoury et al., 2009).

### Molecular biology studies

**Figure 2:**
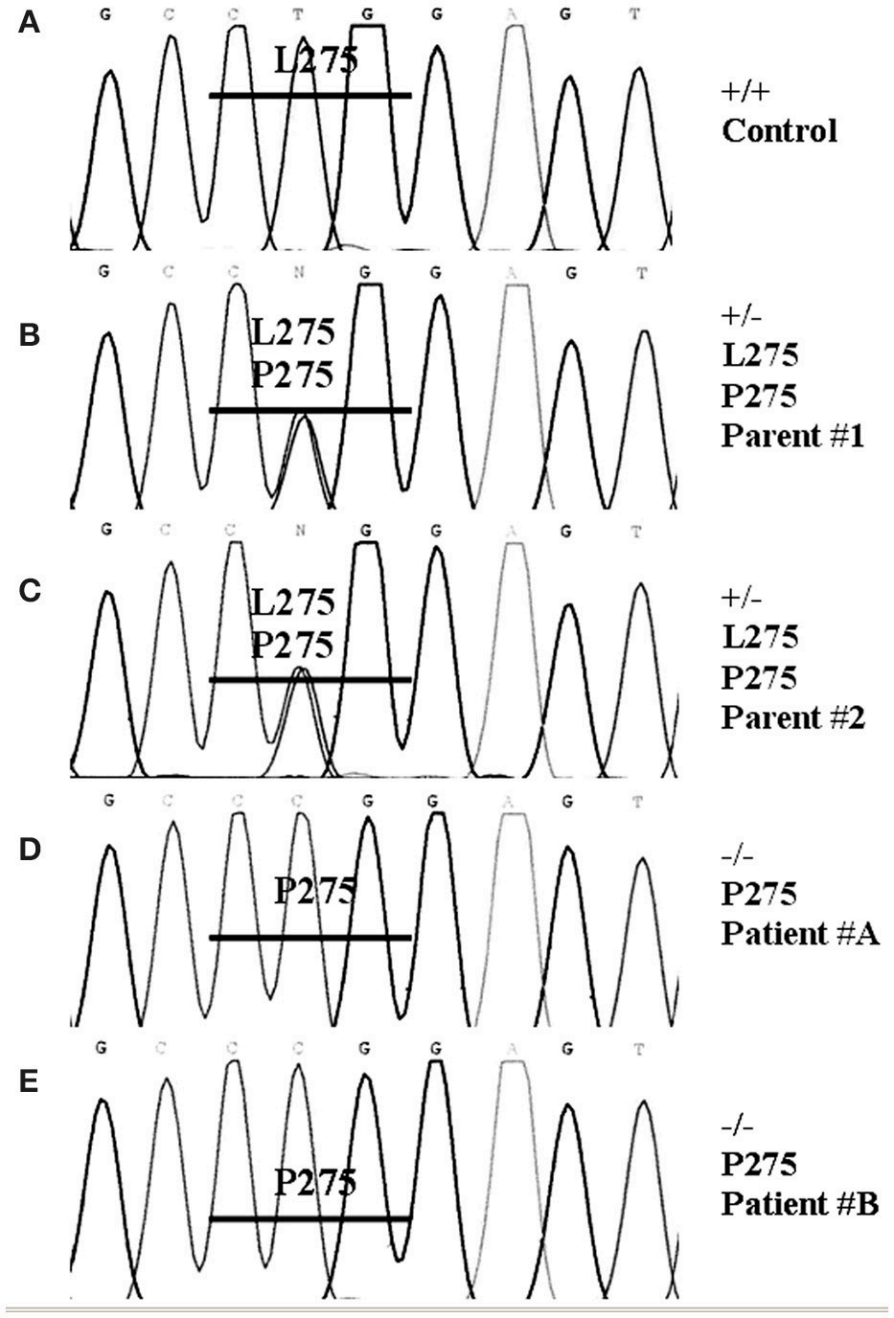
**DNA sequencing**. Genomic DNA was extracted from the blood of two patients, their two parents and a normal individual as control **(A)** using the Invitrogen DNAzol BD reagent. The seven exons of the STARD1 gene were PCR and sequenced. A mutation was found in exon 7. For exon 7 oligonucleotides used for PCR were (5′ to 3′) ATGAGCGTGTGTACCAGTGACG, (5′ to 3′) CCTGGCAGCCTGTTTGTGATAG; the annealing temperature was 60°C and the reaction processed for 30 cycles. The PCR products were sequenced. The mutation found in exon 7 was located at the amino acid residue 275, a leucine being substituted by a proline. The two parents **(B,C)** were +/−, and the two children **(D,E)** were −/−.

Genomic DNA was extracted from the gonadal tissue of patient-A. The seven exons of the STARD1 gene were PCR-amplified using primers and conditions previously described (Bose et al., 1996) and PCR products were sequenced. A mutation was found in exon 7 which was located at the amino acid residue 275, a leucine being substituted by a proline. Genomic DNA was subsequently extracted from the blood leukocytes of the two patients, their two parents and a normal individual. The exon 7 was analyzed for each individual. For the mutation L275P, the control was +/+, the two parents were +/−, and the two children were −/− (Figure 2).

**Figure 3:**
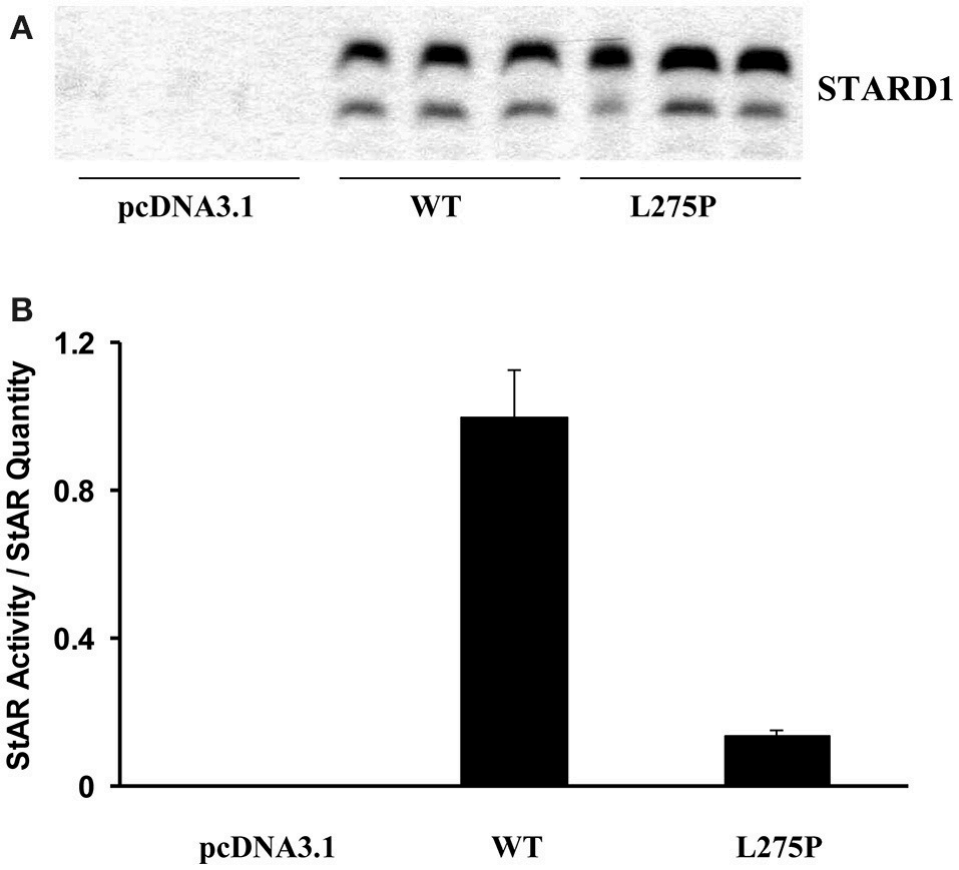
**STARD1 activity and quantification**. COS-1 cells were cotransfected with the F2 construct (Harikrishna et al., 1993) (500 ng) and 200 ng pcDNA3.1 harboring the wild type (WT) human STARD1 cDNA. (WT STARD1-pcDNA3.1) or the mutant L275P STARD1-pcDNA3.1 and 300 ng of pcDNA3.1. Cells were incubated for 24 h. **(A)** 20 μg of whole cell homogenate were analyzed by immunoblotting. The membranes were exposed to anti- STARD1 antibody, followed by a horseradish peroxidase-conjugated anti-rabbit secondary antibody (Fleury et al., 2004). Immunoreactive proteins were visualized with ECL Plus (Amersham Biosciences, U.K. Ltd., Little Chalfont, Buckinghamshire, England) on a Storm 860 laser scanner instrument (Molecular Dynamics, Sunnyvale, CA) and the band intensity was quantified using ImageQuant software. **(B)** Pregnenolone from cell media was analyzed by radioimmunoassay (Fleury et al., 2004). Results are expressed as STARD1 activity/STARD1 quantity after background subtraction.

COS-1 cells were used to determine STARD1 activity and quantification. Figure 3A shows results obtained by western blotting analysis, and Figure 3B shows that the L275P mutant activity was 87% impaired compared to wild type (Khoury et al., 2004).

## Mechanism of cholesterol transfer by STARD1

In order to better explain how clinical mutations affect steroidogenesis, we will discuss the mechanism of action of STARD1 at the molecular level. STARD1 delivers cholesterol from the outer to the inner membrane of mitochondria is poorly understood. STARD1 may act as a sterol transfer protein and enhance sterol desorption from the outer to the inner side of mitochondria (Kallen et al., 1998; Petrescu et al., 2001). In this model STARD1 is directed to the mitochondria via its N-terminus recognition sequence and then, utilizing C-terminal sequences, produces as yet unidentified alterations in the OMM that results in the transfer of cholesterol to the inner side. It is established that STARD1 acts on the OMM and that its level of activity is proportionate to the time it remains at that site (Arakane et al., 1998; Bose et al., 2002).

**Figure 4:**
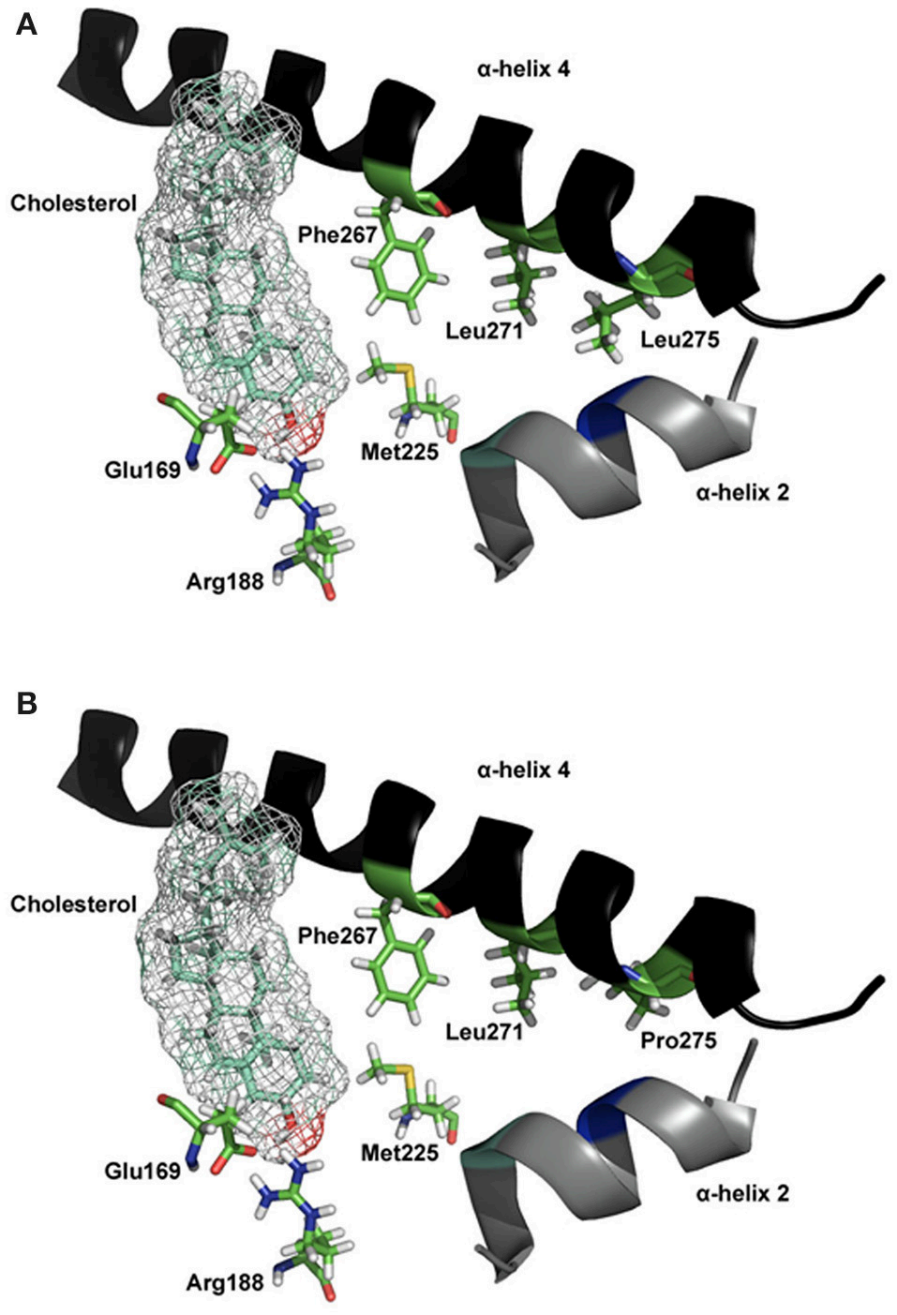
**Molecular model of STARD1 L275P mutation**. **(A)** The essential elements of the wild-type STARD1 are shown. The salt bridge formed by Glu^169^/Arg^188^ putatively interacts with the β-OH of cholesterol, while the α-helix 4 is in a closed state. The line of hydrophobic residues Phe^267^/Leu^271^/Leu^275^ interacting with α-helix 2, Met^225^ and other hydrophobic residues (not shown for image simplicity) help stabilize the STARD1 /cholesterol complex. **(B)** The long hydrophobic side chain for Leu^275^ is absent in the clinical mutation L275P, thereby creates a void in the hydrophobic environment necessary for stabilizing holo- STARD1, leading to a reduction in cholesterol binding and steroidogenic activity (Roostaee et al., 2008). Initial coordinates for the molecular modeling of STARD1 were retrieved from the Protein Databank (code 1IMG). Site-directed mutagenesis and energy minimization were done *in silico* using the molecular modeling software SYBYL 8.0 (Tripos Inc, St. Louis, MO). All the rendering was done using PYMOL (DeLano, W. L. The PyMOL Molecular Graphics System, 2002, DeLano Scientific, Palo Alto, CA).

MLN64, a protein involved in cholesterol transfer in malignant breast cancer, has a significant homology to the C-terminal region of STARD1 and can promote cholesterol transfer (Watari et al., 1997). The C-terminus of STARD1 contains the StAR-Related Lipid Transfer (START) domain which is conserved across a large family of proteins including MLN64 and STARD1 (Ponting and Aravind, 1999; Stocco, 2000; Tsujishita and Hurley, 2000). START domain in MLN64 is homologous to the START domain of the STARD1 protein. Both, functionally similar, are able to bind cholesterol in a 1:1 ratio (Tsujishita and Hurley, 2000; Petrescu et al., 2001). It was first proposed that STARD1 acts as a cholesterol shuttle for transferring cholesterol to the IMM via a hydrophobic tunnel revealed by the crystal structure of MLN64-START (Tsujishita and Hurley, 2000), but the exact mechanism by which STARD1 releases cholesterol to the IMM is unknown (Stocco, 2001). According to computer homology models based on MLN64 structure, STARD1 does not contain a tunnel, but a hydrophobic pocket that can accommodate one molecule of cholesterol (Mathieu et al., 2002a,b; Baker et al., 2005; Yaworsky et al., 2005; Murcia et al., 2006; Lavigne et al., 2010; Thorsell et al., 2011; Létourneau et al., 2014, 2015); the STARD1 crystal structure corroborates this hydrophobic pocket. Several mutations that result in LCAH were mapped onto the MLN64-START structure, in positions residing within the hydrophobic tunnel; these mutations would destabilize the tunnel. In parallel, the residues Glu^169^, Met^225^, and Leu^275^ are part of STARD1's sterol binding pocket and the hydrophobic environment provided by α-helices 2, 3, and 4 (Figure 4). Two regions of STARD1 appear important for the binding of cholesterol and its release during its transfer to the IMM. A cholesterol gating region containing the C-terminal α-helix 4 and a cholesterol binding region buried inside the STARD1 molecule (Roostaee et al., 2008). More specifically, the importance of STARD1's C-terminal region in cholesterol transfer is confirmed by many observations; deletion of 28 amino acids from the C-terminal results in a complete loss of steroids production (Arakane et al., 1996; Wang et al., 1998). This C-terminal deletion corroborates with the truncated STARD1 mutant Q258X and lead to LCAH (Miller, 1997).

*In vitro* experiments have highlighted the role of α-helix 4 as a gate for cholesterol binding (Baker et al., 2005; Roostaee et al., 2008). The helix would be in thermodynamic equilibrium between a partially-unfolded (open) and folded (closed) states (Mathieu et al., 2002a). Upon cholesterol binding, the equilibrium is shifted to the more stable closed state (Baker et al., 2005; Roostaee et al., 2008; Barbar et al., 2009a), until the subsequent release of the ligand by other mechanisms, such as interactions with other mitochondrial proteins, to trigger the transfer of cholesterol from the outer to the inner mitochondrial membrane (Liu et al., 2006; Bose et al., 2008a).

The hypothesis is that the STARD1 C-terminal α-helix 4 movement is a key factor for STARD1-mediated cholesterol transfer inside mitochondria (Mathieu et al., 2002a,b; LeHoux et al., 2003). For instance, biochemical and structural studies of the L275P mutation at the α-helix 4 led to a dramatic reduction in cholesterol binding that correlated with a decrease in the steroidogenic activity of STARD1 (Roostaee et al., 2008).

Several amino acids are in contact with the sterol binding pocket. Figure 4A shows Leu^275^ and other residues forming a hydrophobic core near the end of the C-terminal α-helix 4, and Figure 4B shows the model for the mutation Leu^275^ to proline. Any alterations in these residues, forming a line of hydrophobic residues, would affect the binding and the steroidogenic activity of STARD1. In the case of the L275P mutation, it yielded a 13% pregnenolone production in isolated mitochondria (Roostaee et al., 2008); such a level of steroidogenesis is in agreement with previous studies (Bose et al., 1996; Khoury et al., 2004).

The far-UV CD spectra of STARD1 WT has a 32.5% α-helical content, which is close to the 40% maximum helical content calculated for the three-dimensional model. The L271N and L275P mutants showed a reduced α-helical content of 28.8 and 29.4% respectively compared with STARD1 WT. As discussed above, these mutants were expected to lead to the weakening of the hydrophobic interface and hence to a reduction in the amount of α-helical content and stable tertiary structure (Roostaee et al., 2008). Also, short molecular dynamics simulations have indicated that the L275P mutation confers a higher flexibility to STARD1's α-helix 4 (Barbar et al., 2009b), which suggests a more open state of the helix, thereby reducing its gating capacity, which is related to the decrease in cholesterol binding and the steroidogenic activity previously studied (Bose et al., 1998; Roostaee et al., 2008).

Interestingly, another clinical leucine to proline mutation (L260P) at the α-helix 4 has been found in Swiss LCAH patients and exhibited a similar *in vitro* steroidogenic activity as the L275P mutant (Flück et al., 2005). Hence, one would expect that the L260P mutant might structurally behave in a similar fashion as L275P, by affecting the α-helix 4 gating mechanism.

## Functional activities

Functional activities determined in transfected COS-1 cells revealed differences between different mutants. On the two LCAH patients reported in the study of Lin et al. (1995), the Q258X STARD1 mutant had a functional activity of 17% and the R193X mutant had 14%. Bose et al. reported that the L275P mutant converted 10% more cholesterol to pregnenolone than the vector control (Bose et al., 1996). In 19 Japanese patients, Nakae et al. reported 8 different mutations and in functional studies only the M225T mutation was found to have a partial activity of 44% (Nakae et al., 1997). All other mutants had activities not greater than that of the vector plasmid. STARD1-independent pregnenolone production in the three above mentioned studies was about 14%. Nakae et al. reported on a patient with Q258X, M225T (heterozygous compound) mutations who had a cliteromegaly at the time of diagnosis and moderate elevation of serum testosterone in response to hCG stimulation (Nakae et al., 1997). This patient had the onset of symptoms of adrenocortical insufficiency at the age of 10 months. These authors suggested that late onset of LCAH results from mutations that do not completely inactivate STARD1. The Canadian patient cited by Bose et al. (1996) had the heterozygous compound mutation A218V, L275P, and 46,XY karyotype; her manifestations of corticosteroid deficiency were noted at the age of 2 months.

## STARD1 independence

STARD1 is mainly expressed under acute tropic hormone stimulation. In the absence of STARD1, several elements may account for the basal level of steroid hormone production. For example, MLN64 may be able to enhance the basal low level of steroidogenesis since it is present in human granulosa and theca cells as well as in the fetal adrenal cortex (Watari et al., 1997). Other proteins with a START domain (like STARD4 and D6) might contribute to the basal steroidogenic activity (Soccio et al., 2002; Bose et al., 2008b), but their precise distribution in steroidogenic tissues and role are yet to be clarified. The synthesis or uptake of hydroxysterols might also contribute to the STARD1-independent steroidogenesis; for example, soluble 22R-OH and 25 OH-cholesterol can diffuse to the inner mitochondrial membrane without the participation of STARD1 and be used as substrate for pregnenolone formation (Lin et al., 1995; Roostaee et al., 2008).

## LCAH and STARD1 mutation in canada

In this review, we report the first detailed study of LCAH due to homozygous STARD1 mutation in Canada and, to our knowledge, the first successful pregnancy in a 46,XX female with this disease. Since the discovery of STARD1 in 1994 (Clark et al., 1994), many important works contributed to increase our understanding on the role played by this protein in the steroidogenesis and the physiology of LCAH. We have identified a homozygous recessive mutation on the STARD1 gene of our two patients and the heterozygous mutation in their mother and father. This mutation resulted in the substitution of a leucine by a proline (L275P) in the STARD1 protein (Khoury et al., 2004), which did not conserve more than 13% of its steroidogenesis activity compared to wild type STARD1, as analyzed in COS-1 cells. This is in agreement with Bose et al. (1996) who reported that the mutated L275P STARD1, found in another Canadian, converted 10% more cholesterol to pregnenolone than the control vector. The Canadian patient cited by Bose et al. (1996) had the heterozygous compound mutation A218V, L275P, and 46,XY karyotype; her manifestations of corticosteroid deficiency were noted at the age of 2 months. We did not have the opportunity to obtain a genetic history for this patient; we know however (personal communication from Dr. Rose Girgis, Edmonton, Canada) that the parents are from French Canadian origin who lived in the province of Quebec before moving to the west. It is unknown if any parental connection exists between these two families who share the allele L275P. Although the number of patients bearing the L275P mutation is too small to be conclusive, it might indicate a possible founder effect for this disease in Canada (Bose et al., 1996).

## Clinical manifestation and efficiency of cholesterol transfer system

The two patients, A and B, described in this study had a normal health before their first admission to the hospital for severe manifestations of mineralocorticoid deficiency and hyperpigmentation at the age of 11 and 4 1/2 months respectively. Some residual secretion of aldosterone was present and basal blood cortisol was in the low normal level, but without any significant response to ACTH stimulation up to the age of 3 years for patient A and only 15 months for her sister. Consequently, patient A seemed to have less severe corticosteroid deficiency than patient B and both patients have less severe mineralocorticoid and glucocorticoid deficiencies than reported in similar cases (Bose et al., 1996).

To date, at least 97 patients with LCAH and more than 35 different mutations have been reported in the STARD1 gene (Bhangoo et al., 2005, 2006; Papadimitriou et al., 2006; Abdulhadi-Atwan et al., 2007). Two distinct genetic clusters were initially reported: More than 70% of Japanese alleles and all Korean alleles carry the Q258X mutation; a second genetic cluster is found among Palestinian Arabs, most of whom carry the R182L mutation. Many publications underline that some correlations exist between the severity of STARD1 mutation and the age of onset of a clinical salt-wasting crisis, hypoglycemia and gonadal function (Bose et al., 1996; Miller, 1997; Bhangoo et al., 2005, 2006). Patients with the mutation Q258X or R182L are generally symptomatic by the age of 1 day to some weeks. Three unrelated patients of Swiss ancestry, L260P mutant, had their onset of symptoms at the age 2.5–5.5 months (Flück et al., 2005). Furthermore, as reported by Chen et al. (2005) patients from eastern Saudi Arabia who carry the R182H mutation, very close to the Palestinian mutation R182L, had a milder disease starting at the age of 1–14 months, and 4 out of 7 of them had the onset of their symptoms from 7–14 months of age; functional study in this group showed a complete loss of STARD1 activity. One patient from Qatar, with R182H mutation, had the onset of clinical symptoms and laboratory evidence of salt loss at the age of 3 weeks (Achermann et al., 2001); the *in vitro* STARD1 activity of this patient was not determined. The patients reported by Chen et al. (2005) as well as our two patients raise the question about the different clinical manifestations and time of onset with the same genetic mutations, in the same ethnic group or family members and the same geographic region. There is, clearly, a limited sensitivity of the different functional assays for determination of STARD1 activity which may explain the poor correlation frequently observed between the clinical findings and the results of *in vitro* studies. There is also a wide spectrum of heterogeneity in the clinical manifestations and biochemistry, as well as, the time of onset of different symptoms in LCAH. However, in our case, since the assays for Patients A and B are relatively standardized, the sensitivity is the same.

Interestingly, both patients carry the same mutation, yet the clinical manifestation of LCAH was delayed between them. The physiological conditions are different from one pregnancy to another and the demand for different steroids is variable during fetal development. Indeed, during pregnancy, conditions such as nutrition, hydration/dehydration, disease and other stress, may have an influence on steroidogenesis. In addition, the circumstances of delivery and the neonatal environment may have different patterns of tropic hormone stimulation which may modulate the extent of acute steroidogenic demands.

The STARD1 -dependent and STARD1 -independent steroidogenesis seem to be good descriptive models to explain the chronology of events in non-placental steroidogenic tissues. It is not clear however what determines the length of time an infant with LCAH can survive before experiencing a salt-wasting crisis (Hauffa et al., 1985; Fujieda et al., 2003; Bhangoo et al., 2006). We still need more information concerning the function or loss of function of the mutated STARD1 and what determines the longevity and efficiency of the STARD1 -independent system. It is clear, however, that early appeal and continuous stimulation of steroidogenesis in the gonads (by the hormones hCG, FSH, LH) and the adrenals (by ACTH and the renin-angiotensin system) are the starting point initiating the failure of both systems. In this study, our two patients, Patient A, 46,XY and Patient B, 46,XX, members of the same family with the same homozygous (L275P) mutation, have different severity of the disease and different time of onset of corticosteroid deficiency. Clinical manifestations and hormonal data prove the severe deficiency of testosterone secretion in Patient A, the milder ovary dysfunction in Patient B and the presence of some residual corticosteroid secretion and progressive post natal adrenal failure for both.

## Gonadal function in LCAH and 46,XY genotype

Patient A in this study is a 46,XY genotype with female external genitalia and blind vaginal pouch. Figure 1 shows lipid accumulation in Leydig cells typical of LCAH. The absence of androgen secretion, basal and after hCG stimulation, as well as the absence of Wolffian structures testify on the early and severe deficiency of testosterone secretion necessary for differential sexual development and virilization. In fact, during the early weeks of embryonic development, the fetus is exposed to acute hCG stimulation which may lead to lipid accumulation in the cell due to the lack of STARD1 activity, thereby destroying Leydig cells. This is clearly contrasting with the relatively moderate severity of mineralocorticoid and glucocorticoid deficiency of this patient. Generally, all 46,XY reported patients had a profound impairment of testosterone synthesis (Bose et al., 1996), however Wolffian duct derivatives are sometimes well developed as reported by Nakae et al. (1997) on patient 7 (Q258X, M225T) who had a cliteromegaly and moderate secretion of testosterone in response to hCG stimulation.

This seems to be concordant with the hypothesis of the earlier mentioned STARD1-dependent steroidogenesis deficiency and STARD1-independent impairment of steroidogenesis due to Leydig cells destruction following lipid accumulation caused by an early and continuous stimulation (placental hCG, fetal pituitary LH). Such patients should be raised as females (Bose et al., 1996) and should undergo orchidectomy due to the risk of *in situ* testicular carcinoma development (Korsch et al., 1999).

## Gonadal function in LCAH and 46,XX genotype

### Spontaneous puberty

Patient B in this study is a unique case. While she had an earlier and more severe corticosteroid deficiency than her sibling, her ovarian function was less disturbed than what was observed in other similar cases (Bose et al., 1996). By the end of 1990, at the age of 11 7/12 years, she started a progressive pubertal development; her ovarian response to hCG stimulation was normal and she had menarche at the age of 14 2/12 years (Khoury et al., 2004). This was unexpected for LCAH in 1993. Our observations are in agreement with Matsuo's report in 1994 (Matsuo et al., 1994) that 5/5 46,XX patients with LCAH aged more than 13 years expressed a spontaneous development of secondary sex characters and vaginal bleeding at the time of puberty, their estrogen level ranging from 22–85 pg/ml. Bose et al. (1997) described in 1997 the first genetic analysis in a 46,XX female homozygous for STARD1 shift mutation 261ΔT and spontaneous feminization. Three other cases were reported by Fujieda et al. (1997, 2003)who found that the ovaries of such patients were enlarged in the post pubertal stage with many cysts occupying the entire volume of the ovary and with hypertrophied ovarian stroma. This phenomenon was not observed on repeated echographies in our Patient B. The heterogeneity in the pubertal development of 46,XX female patients with STARD1 mutation was also described by Nakae et al. (1997) who reported on 6/10 46,XX patients who experienced spontaneous pubertal changes, irregular menstruations, anovulatory cycles and in some a polycystic aspect of ovaries. All these reports show that the difference in the extent of impairment of the testis and ovaries is dramatic in LCAH. However, although initially unexpected, even the most severely affected 46,XX female patients undergo spontaneous feminization, breast development and cyclical vaginal bleeding at the usual age of puberty (Matsuo et al., 1994; Bose et al., 1997; Nakae et al., 1997).

The fetal ovary cells are quiescent and in affected 46,XX patients these–cells should remain undamaged since they should not accumulate cholesterol esters until they first undergo gonadotropin stimulation. This would explain why these cells retain steroidogenic capacity and hence at the time of puberty can make estrogens, albeit, in subnormal amounts, resulting in high gonadotropin secretion. However, only the cells in the individually recruited follicles undergo stimulation, and hence only these cells accumulate cholesterol esters and lose steroidogenic capacity. Regular monthly cycles are possible, because a large number of follicles remain relatively undamaged before recruitment. Such monthly cycles, which may persist for years, are probably anovulatory and can produce a large quantity of ovarian cysts.

## STARD1 expression in the ovaries

The study of Sandhoff and McLean (1996) shows that in rat ovaries STARD1 mRNA expression is controlled by tropic hormones PMSG (post-menopausal stimulating gonadotropin) and hCG. hCG increases the steady-state level of STARD1 mRNA in the ovary during follicular and luteal development. The rise in STARD1 expression paralleled the rise in serum progesterone levels, consistent with STARD1's presumed role in the regulation of steroidogenesis. Furthermore. STARD1 gene expression in primary cultures of porcine granulosa cells is stimulated above control levels by FSH, 8-bromo-cAMP and IGF-1 respectively. FSH and IGF-1 interact synergistically to induce amplification of STARD1 mRNA and protein expression in serum-free monolayer culture of immature swine granulosa cells (Balasubramanian et al., 1997). During follicular development in the rat ovary, Ronen-Fuhrmann et al. (1998) have examined the time-dependent expression of STARD1 mRNA and protein in PMSG/hCG-treated immature rats. They found that in the early phase, before the administration of tropic hormones, granulosa cells did not express STARD1. The first peak of STARD1expression was generated by PMSG administration. The expression was restricted to the ovarian secondary interstitial tissue as well as to a fewer scattered theca interna cells producing androgens which can synergistically potentiate FSH-induced actions in granulosa cells, and P450 aromatase for estrogen production in granulosa cells of Graafian follicles. This also seems to initiate the transition of follicles from the small antral stage to the preovulatory stage. After hCG administration, both the granulosa (until now devoid of STARD1) and the theca-interstitial cell types joined in synchronized production of STARD1. At this phase of follicular development, usually perceived as the onset of the luteinization process, high STARD1mRNA levels resumed in the all-ovarian interstitium and high levels of STARD1were also expressed in the granulosa cells; however, in the later cell type, STARD1 expression was strictly confined to periovulatory follicles.

## Induction of ovulation

The measurement of blood FSH, LH, E2, and other hormones, at different ages in patient B, argues for a normal function of the hypothalamus-pituitary axis with a progressive increase of 17β-estradiol secretion to induce progressive pubertal feminization and regular menstruations. This was probably due to an efficient mechanism of STARD1 -independent steroidogenesis still undamaged by the toxic accumulation of cholesterol and its derivatives, during the short life of stimulated follicles. However, data showed that she failed to demonstrate the spontaneous estrogen surge of the end of follicular phase, necessary for the secretion of ovulatory peak of LH, ovulation and consequently the normal increase of progesterone and basal temperature during the next theoretical luteal phase. The menstrual cycles of this patient were anovulatory for many years before consulting for infertility problems. Interestingly, during her early pubertal development, a hCG stimulation test resulted in a small increase in androstenedione and substantial enhancement of 17β-estradiol secretion (Khoury et al., 2009).

The mechanism of this response is subject to speculation, but more importantly it indicates that the steroidogenic systems retained some capacity to respond to tropic hormones. Thus, during the follow-up in the fertility clinic, we proposed to observe the function of the hypothalamus-hypophyso-ovarian axis during a Clomiphene stimulation test. This was done at the age of 25 years. Endocrine data shows that pituitary gonadotrophins, induced by Clomiphene stimulation, enhanced the steroidogenesis process in the granulosa cells, in the same manner as was observed with the hCG stimulation test, 13 years earlier. Furthermore, the high levels of progesterone and 17β-estradiol observed during the second half of her menstrual cycle is a clear confirmation of a process of ovulation and luteinization (Khoury et al., 2006b, 2009). According to Ronen-Fuhrmann et al. (1998) this is due, in the normal rat, to the high STARD1 expression induced by tropic hormones in all ovarian interstitium and the granulosa cells of periovulatory follicles. In our patient, this may indicate that hCG stimulation or increased secretion of pituitary FSH and LH induced by Clomiphene, as well as the ovulatory peak of LH, may have a role of further activation of the STARD1-independent system (Watari et al., 1997). It is also plausible to think that the amplification of the mutated STARD1 expression by those tropic hormones may contribute, via its partial steroidogenic activity, to the production of estrogen surge necessary for the ovulatory peak of LH and ovulation.

## STARD1, corpus luteum, and pregnancy

Pregnancy is the ultimate proof of the induction of ovulation. In our patient this was produced by Clomiphene stimulation. We believe that any other protocols of ovulation induction by gonadotrophins will also have a good chance of success. Our patient had a first pregnancy at the age of 25 4/12 years. However, a spontaneous abortion occurred 6 weeks later. The human corpus luteum derived from the ovulated follicle is an active producer of progesterone, an essential hormone for establishing and sustaining early pregnancy (Carr, 1992). Placental progesterone becomes sufficient to maintain pregnancy only after about 6 weeks of gestation, the so-called “luteoplacental shift.” Consequently, human maternal ovariectomy in the first 6 weeks will cause spontaneous abortion, but maternal ovariectomy thereafter will not (Csapo et al., 1973; Csapo and Pulkkinen, 1978). The critical step in luteal progesterone secretion is the movement of cholesterol from the OMM to the IMM (Devoto et al., 2002). Examination of corpora lutea of different luteal phases revealed that the basal expression of STARD1 transcript and protein was greatest in early and mid-luteal phase to decline in the late-luteal phase. Furthermore, under hCG stimulation, the expression of the major 1.6 kb STARD1 mRNA transcript is rapidly enhanced. The rise in STARD1 expression paralleled an increase in progesterone levels (Chung et al., 1998; Devoto et al., 2001). In human corpus luteum, the theca-lutein cells and granulosa-lutein cells exhibited marked heterogeneity in STARD1 protein concentration, with theca-lutein cells expressing greater levels of STARD1 than granulosa-lutein cells, irrespective of the stage of the luteal phase (Devoto et al., 2001; Christenson and Devoto, 2003). Human chorionic gonadotropin treatment during the late-luteal phase causes a pronounced increase in both theca-and granulosa-lutein cell STARD1 gene expression. In some species, including human, PGF2α is believed to be the physiological agent responsible for causing corpus luteum regression at the end of a non-fertile cycle. This luteolytic compound has been shown to cause a pronounced decline in STARD1 gene expression, altering cholesterol transport to the mitochondria, inhibiting progesterone production and leading to substantial stores of lipids (Devoto et al., 2001; Christenson and Devoto, 2003). The distribution of STARD1 inside theca and granulosa-lutein cells of human corpus luteum was assessed by electron microscopy (Sierralta et al., 2005). Greater levels of STARD1 immunolabeling was found in the cells from early- and mid- than in the late-luteal phase of corpus luteum, and lower levels in cells from women treated with GnRH antagonist in the mid-luteal phase. There is also a substantial amount of mature STARD1 protein (30 kDa) in the cytoplasm of luteal cells (Sierralta et al., 2005). The presence of STARD1 in the cytoplasm was also reported in rat adrenal homogenates (LeHoux et al., 1999). The administration of GnRH antagonist during mid-luteal phase causes a dramatic reduction in STARD1 immunolabeling in both the cytosol and mitochondria of theca and granulosa-lutein cells of mid-luteal corpus luteum suggesting that, the levels of STARD1 protein in both cell compartments are LH dependent. Also, the presence of MLN64 in the supernatant of human corpus luteum extract throughout the luteal phase (Watari et al., 1997; Kishida et al., 2004) supports the idea that in cells with high steroidogenic activities, proteins with a START domain, other than STARD1, might participate in the intracellular trafficking of cholesterol or other lipids.

We conclude that the early spontaneous abortion at the first pregnancy was due to a dysfunction of this mutated STARD1 inside the theca and granulosa cells of the corpus luteum. The STARD1-dependent steroidogenesis was highly solicited during the early and middle phase of the corpus luteum development by an acute and important stimulation of hCG. The incapacity of the mutated STARD1 to transport the high quantity of cholesterol to the IMM will result in an accumulation of cholesterol, cholesterol esters, and oxidative products in the steroidogenic cells of the corpus luteum. This leads to the destruction of both the STARD1-dependent and STARD1-independent systems, in agreement with the two-hit theory concerning STARD1mutations in steroidogenic tissues. Moreover, this explains the failure of the corpus luteum to produce the progesterone necessary for preparing and stabilizing the endometrium, and protecting the embryo during the early first trimester (Christenson and Devoto, 2003; Sierralta et al., 2005). Substitution therapy with progesterone was then indicated. It was recommended to start progesterone at the 17th day of the cycle and to continue this treatment, during the first trimester, if pregnancy was confirmed later. This technique was successful since a second pregnancy occurred. Progesterone treatment was initially recommended throughout the pregnancy hoping to optimize uterine quiescence and prevent premature delivery in her multiple gestations (Meis, 2005); but for some reason, it was stopped at 25 weeks. Delivery of two normal boys occurred at 30 weeks of gestation. The same treatment was used for a third pregnancy, however progesterone administered up to the 17th week was deemed sufficient to support a single gestation. This led to the delivery of a normal girl at 36 weeks of gestation (October 2008).

In 2008, Sertedaki et al. reported a female LCAH patient bearing a 11-bp deletion in exon 6 of her STARD1 gene (Sertedaki et al., 2009). She did have “cysts in the ovaries” and the LH/FSH ratio was very high. After many unsuccessful attempts to conceive, she entered a program of IVF. Follicle development and growth was easily obtained by the administration of recombinant FSH and recombinant LH and follicle maturation was initiated by hCG administration before oocyte retrieval. After IVF and implantation, vaginal administration of progesterone was conducted to support pregnancy up to the 11th week. A female baby was born by cesarean section in 2007 with no perinatal complications. The used protocol was a classic strategy for IVF. Furthermore, it is fully concordant with our strategy for induction of ovulation and protecting pregnancy in LCAH during the first trimester of gestation. In our case, we used Clomiphene for ovulation induction and only 400–600 mg/d of progesterone (Prometrium) (oral and/or vaginal) was given (Smitz et al., 1992; Progesterone supplementation, 2008).

## Pregnancies observations

Ovulation induction with Clomiphene citrate is associated, in 6–8 % of cases, with multiple pregnancies, mainly twins (Messinis, 2005). During her last two pregnancies, the patient B developed high blood pressure. Because it first started during pregnancy and it returned to normal after delivery, we classified it as gestational hypertension. She never met preeclampsia criteria. The onset of high blood pressure was earlier in the second pregnancy (16 + 6 weeks GA) than in the third one (34 weeks GA), perhaps because of the twin pregnancy status which represents a risk factor (Creasy et al., 2004).

We do not know exactly the cause of the two preterm deliveries preceded by premature preterm rupture of membranes (PPROM). Although high dosages and regular daily oral steroid administration during pregnancy has been associated with preterm delivery and PPROM (Laskin et al., 1997), this was probably not the case for our patient who received physiological doses of hydrocortisone and fludrocortisone acetate during her pregnancies. Furthermore, in this case, we not believe that there is an association between preterm delivery from PPROM and cessation of progesterone, since the patient did not complain of premature contraction or significant uterine activity before the delivery. Also, stimulation with oxytocine has been necessary for delivery. Finally, even if infection is a main causative agent in PPROM, she didn't have any criteria associated with that.

## Conclusion

We have described clinical data collected over 38 years of follow-up on two French-Canadian patients with 46,XY and 46,XX karyotype and presenting the classical clinical manifestations of LCAH due to a homozygous STARD1 mutation (L275P).

The two patients presented the classic picture of LCAH. Mineralocorticoids and glucocorticoids deficiencies are less severe in Patient A than Patient B. Both patients have had less severe manifestations of corticosteroid deficiency and a later onset of clinical symptoms than the vast majority of patients suffering from STARD1 mutations described in the literature. Patient A (46,XY) portrayed clinical and biochemical signs of early and severe deficiency of testosterone secretion. Testosterone is necessary for differential sexual development and virilization in the early weeks of fetal life during the period of high stimulation by hCG.

Patient B, 46,XX karyotype, is probably one of the first patients with LCAH in which spontaneous pubertal development was observed. She had menarche and spontaneous regular non-ovulatory menstruations for many years before consulting the fertility clinic. hCG and Clomiphene stimulation tests proved the possibility of increasing estrogen secretion by an acute stimulation of the ovarian theca interstitia and the granulosa cells. Clomiphene stimulation restored the surge of 17β-estradiol and LH secretion of the mid-cycle to induce ovulation and open the door for pregnancy. Our understanding of the physiological role of STARD1 during the different phases of the corpus luteum was essential to explain the miscarriage at 6 weeks of the first pregnancy, and then to develop a preventive therapy by progesterone administration during the first trimester of the following pregnancies. We were able to describe and present the first case of pregnancy in the patient with LCAH. It was a very long way from a fatal disease to a normal healthy life and restoration of the reproductive function in a female with the L275P mutation of STARD1.

The clinical and biochemical data of our two patients are concordant with the STARD1-dependent and STARD1 -independent mechanisms of steroidogenesis. However, the non-STARD1 steroidogenesis system is, in our opinion, the basal physiologic system of steroid synthesis producing normal adrenal and gonadic steroids during the normal physiological circumstances. Furthermore, the STARD1 -dependent steroidogenesis system seems to be a key rate-limiting mediator in the acute regulation of steroidogenesis by tropic hormones. It is acting during the acute need for more production of steroids during physiologic periods of normal development (as sexual differentiation and virilization in the early fetal life of the male), surgical or infection stress, normal surge of estrogen production in the mid-cycle necessary for ovulation, or the high production of progesterone during the first trimester of pregnancy. The incapacity of the mutated STARD1 to assume this high speed function, during stress situations and tropic hormone stimulation, leads to the accumulation of high levels of cholesterol, cholesterol esters and oxidative products in the cell. Early appeal and continuous stimulation of steroidogenesis in the gonads (by the hormones hCG, FSH, LH) and the adrenal (by ACTH and renin-angiotensin system) are the starting points initiating the failure of both systems of steroid production.

**Figure 5:**
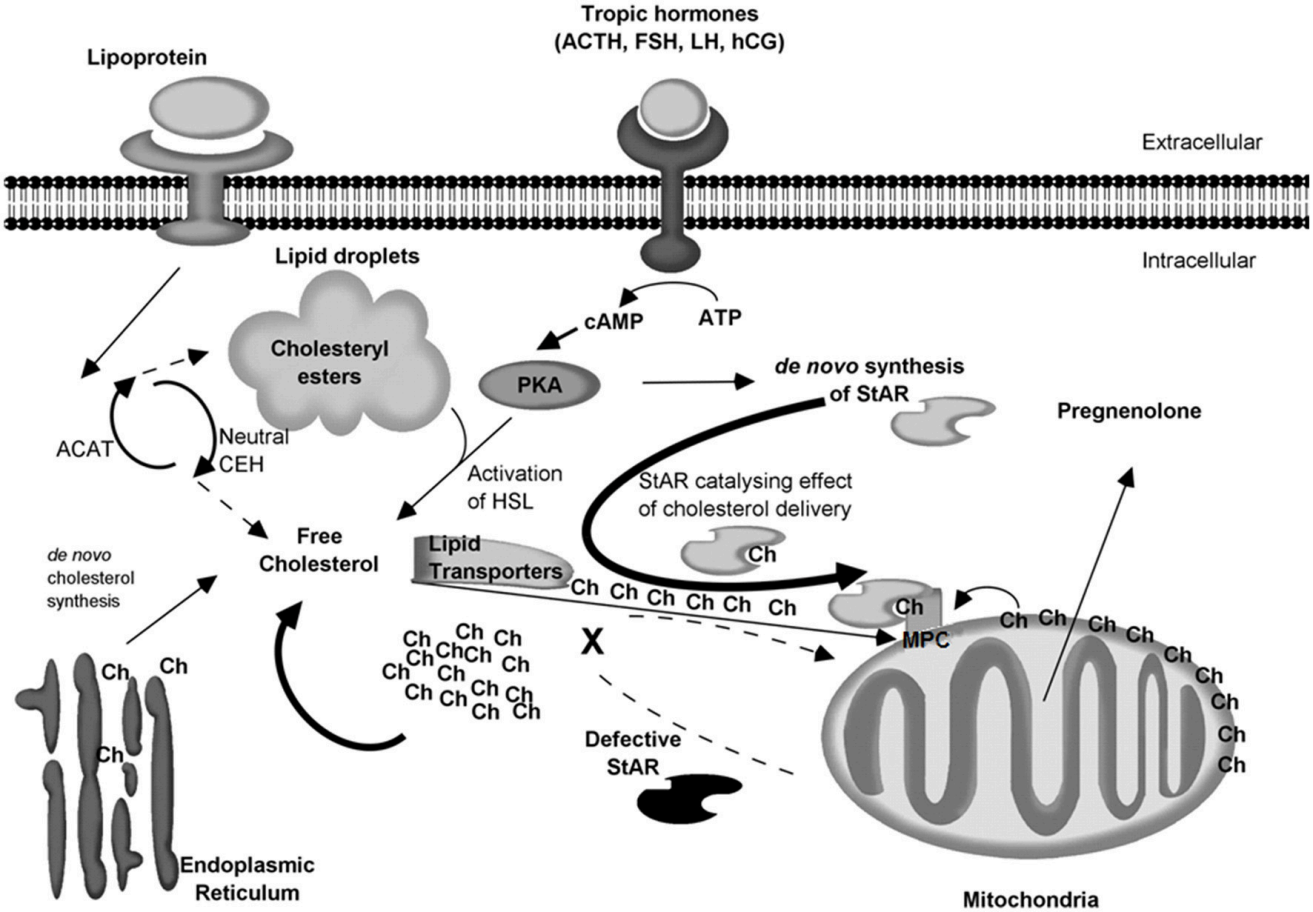
**Cellular model of steroidogenesis and LCAH**. Low and high density lipoproteins (LDL and HDL) are captured by receptors on the cell membrane. Initial metabolism of LDL lipids occurs in the lysosome where cholesteryl esters are hydrolyzed by acidic cholesteryl ester hydrolases (acidic CEH). The resulting free cholesterol (Ch) is re-esterified by the cytosolic acyl-CoA:cholesterol acyltransferase (ACAT) and stored in lipid droplets. Cholesterol can also be synthesized *de novo* in the endoplasmic reticulum. In the resting cell, cholesterol is constantly hydrolyzed/re-esterified by neutral CEH and ACAT. With the help of lipid transporters, free cholesterol can be conveyed at the OMM. Then cholesterol present at the OMM can be transferred to the IMM for conversion to pregnenolone without STARD1. This *low capacity* system may explain the basal level of hormone production (10–13%) for homeostasis. Following an acute event (stress, dehydration, puberty, etc.), tropic hormones stimulate steroidogenic cells and activate PKA, which in turn leads to three actions: The activation of the hormone-sensitive lipase (HSL) which releases cholesterol from lipid droplets, the *de novo* synthesis of STARD1, and the formation of a multiprotein complex (MPC) somehow involved in the transfer of cholesterol to the IMM (Liu et al., 2006; Bose et al., 2008a). Then STARD1 may act as a *high capacity* system by catalyzing the delivery of cholesterol to the OMM and the MPC complex for its transfer to P450scc in response to the acute demand for steroid hormones. In the case of LCAH, the STARD1 high capacity system is impaired cannot support the substantial throughput of cholesterol, and the latter accumulates in the cytosol; lipid droplets become engorged with cholesterol. As lipid droplets accumulate, the cell becomes less functional and LCAH is onset.

Figure 5 shows an up to date model of steroidogenesis and LCAH. Despite all the progress made during the last 20 years in the physiology of the transfer of cholesterol to the IMM (Liu et al., 2006; Bose et al., 2008a; Miller, 2016), we still need to understand the detailed interplay between STARD1 and its delivery of cholesterol to a multiprotein complex (MPC) (Liu et al., 2006; Bose et al., 2008a) somehow involved in the transfer of cholesterol to the IMM.

Finally, we wish to point the reader to our recent progress on the dynamics and mechanism of ligand binding and release of the START domain of STARD6 (Létourneau et al., 2016).

## Author contributions

KK was in charge of patients. EB and PL contributed to figures. JGL, EB, and KK contributed to the writing of this article. YA and AO did the clinical follow-ups of the pregnancies.

### Conflict of interest statement

The authors declare that the research was conducted in the absence of any commercial or financial relationships that could be construed as a potential conflict of interest.

## Abbreviations

StAR: steroidogenic acute regulatory protein (STARD1)
LCAH: lipoid congenital adrenal hyperplasia
P450scc: cytochrome P450 side chain cleavage
Testo: testosterone
Andro: androstenedione
DHEA: dehydroepiandrosterone
Prog: progesterone
E_2_: 17β-estradiol
LH: luteinizing hormone
FSH: follicular stimulating hormone
17 OH-Prog: 17 hydroxy-progesterone
17 OH-Preg: 17 hydroxy-pregnenolone
ACTH: adrenocorticotropin
hCG: human chorionic gonadotropin
PRA: plasma renin activity
PCR: polymerase chain reaction
PMSG: post-menopausal stimulating gonadotropin
IVF: *in vitro* fertilization
PPROM: premature preterm rupture of membranes.
